# Effects of Solidification Pressure and Heat Treatment on the Microstructure and Micro-Hardness of AlSi9CuMg Alloy

**DOI:** 10.3390/ma12142229

**Published:** 2019-07-10

**Authors:** Lijun Wei, Baoshuai Han, Fan Ye, Yanjin Xu, Sujun Wu

**Affiliations:** 1 School of Material Science and Engineering, Beihang University, Beijing 100083, China; 2 Material Department, AVIC Manufacturing Technology Institute, Beijing 100024, China

**Keywords:** squeezing casting, high pressure, melt flow, solid solution, strengthening

## Abstract

The effect of high pressure (135 MPa) and the following heat treatment on the microstructure and micro-hardness of the squeezing cast AlSi9CuMg alloy is investigated, using optical microscopy (OM), Vickers tester, scanning electron microscopy (SEM), and transmission electron microscopy (TEM). The results indicate that the application of high pressure can increase under-cooling and the cooling rate during solidification and cause the refinement of the microstructure. The enhanced melt flow resulting from high pressure can also break the dendrite to form the spherical and elliptical primary α (Al) grains during the early stage of solidification. The winter-sweet flower-shaped primary α (Al) phases can also be formed through plastic deformation caused by the flow of the partially solidified melt. The ageing treatment results showed that a maximum (peak) micro-hardness value was obtained for each of the three ageing temperatures at different ageing times, and the highest peak value was achieved at 175 °C for 480 min. The micro-hardness change of the sample under different ageing processes was attributed to the variation of type, density, and size of the precipitates.

## 1. Introduction

Al–Si alloys are widely used in the automobile industry due to their excellent comprehensive mechanical properties and low density [1,2,3,4]. The mechanical performance depends sensitively on the microstructure and casting defects, which are controlled by casting methods [5,6]. Die casting, in which molten metal solidifies under high pressure, is widely employed in the manufacture of components with the advantages of no porosity, high mechanical properties, good surface smoothness, and accurate dimensional precision [7,8,9]. In the conventional die casting process, porosities are caused by gas entrapment resulted from the high-speed filling of the liquid metal. Bubbles may be formed at the casting surface during heat treatment due to the expansion of the gas in the pores, which are detrimental to the surface quality and mechanical performance [10]. To avoid the formation of the bubbles at the surface, squeezing casting with a low filling speed, which can effectively expel the gas and eliminate the pores, has been proposed and adopted to fabricate some structural safety components.

The microstructure and molten mobility can be improved by the application of high pressure. As investigated by Ashiri et al. [5] and Bai et al. [11], the microstructure of Ai–Si alloys was composed of the fine dendrite and eutectic silicon at a same pouring temperature of gravity casting. In addition, the composition of the eutectic point would move toward the Si direction and the eutectic temperature presents an ascendant tendency with the increase of casting pressure [7]. With a lower pouring temperature than in the normal casting process, casting porosities can be eliminated effectively by squeezing casting due to the forced feeding caused by high pressure. According to previous research, the shape of most α (Al) grains would change from dendritic to globular when formed by near liquidus squeezing casting. However, there is severe segregation and size difference among the primary α (Al) phase [12,13].

The effect of microstructure and casting defects on mechanical performance has been extensively studied in numerous investigations. The mechanical properties of Al–Si alloys depend greatly on the shape and size of secondary phase and casting defects, such as eutectic silicon, iron-rich phase, microvoids, and shrinkage holes [14,15,16]. The anisotropic morphology of secondary phases can induce some serious stress concentration, and cracks may initiate at the interface of secondary phase and primary α (Al) even in the low-strain region [17]. Generally, the fracture of Al–Si alloys can be caused by the cracking and debonding of those secondary phases [10]. The large eutectic Si phases will become the favorable sites for the formation of cracks under tensile loading. Therefore, superior mechanical properties can be obtained with the fine spherical Si particles [18].

The effect of high solidification pressure on microstructure and mechanical performance has been investigated in numerous studies. Results showed that dendritic grains were obtained through squeezing casting at a high pouring temperature (720 °C) [11], while severe macro-segregation would occur in the component produced by squeezing casting at a relatively low pouring temperature (lower than 660 °C) [12]. However, there are few studies performed with the pouring temperature between these two. In the present work, the component was fabricated by squeezing casting at a pouring temperature of 680 °C and was then subjected to solid solution treatment at 535 °C for different holding times. The corresponding microstructure and micro-hardness were investigated and are explained in detail.

## 2. Materials and Methods

**Figure 1:**
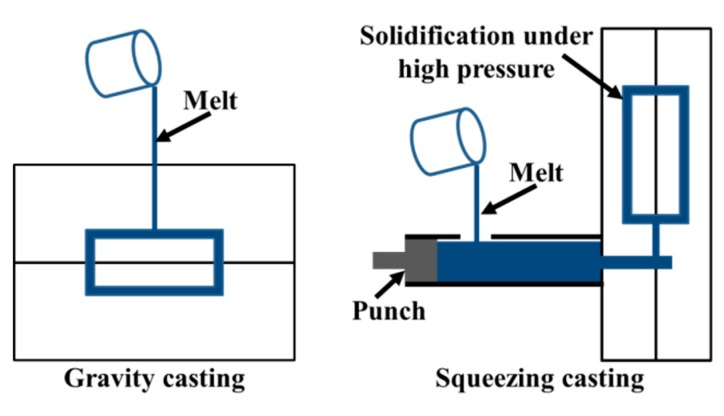
Schematic diagram of gravity casting and squeezing casting.

The chemical composition of the alloy used in the research is shown in Table 1. The components were fabricated through squeezing casting and gravity casting, respectively; the schematic diagrams are shown in Figure 1. The alloy was prepared from high purity Al, high purity Mg, Al–30Si, Al–50Cu master alloys. Before pouring, the alloy was melted in an electrical furnace and held for 10 min at 720 °C. The refining agent was added into the melt and stirred for 5 min. Subsequently, the molten alloy was transferred into a holding furnace. Gravity casting was performed with a pouring temperature of 680 °C and mold temperature of 250 °C. Squeezing casting was conducted by an indirect squeezing casting machine (Impress III280D, Lijin, Hongkong, China). Firstly, the melt was poured into the filling chamber. Subsequently, the molten melt was injected into the mold with a filling velocity of 0.1 m/s. In order to eliminate the gas and shrinkage porosity, it is necessary to increase the solidification pressure with the increase of component thickness. According to previous experiment results, the solidification pressure of 135 MPa was selected and applied immediately when the mold filling was finished in this study. The squeezing casting process was performed at the same pouring and mold temperatures as the gravity casting.

In order to study the effect of heat treatment on microstructure, the samples taken from the components were subjected to solution treatment at a temperature of 535 °C for 15 min, 30 min, 45 min, 60 min, 120 min, 180 min, and 240 min. In addition, to elucidate the effect of ageing treatment on hardness, the ageing treatment was performed at 160 °C, 175 °C and 190 °C for different time periods.

The characterization of the microstructure was performed through optical microscopy (OM, Leica DMLM, Buffalo Grove, USA), scanning electron microscopy (SEM, Zeiss SUPRA 55, Munich, Germany), electron probe micro-analysis (EPMA, Shimadzu EPMA-1720, Sichuan, China), and transmission electron microscopy (TEM, JEM2100F, Akishima, Japan). The samples for OM and SEM were polished and etched for 3 s using a solution composed of 2.5% HNO_3_+1.5% HCl+1.0% HF+95% H_2_O before microstructure observation. The EPMA samples were polished without the following etching process. TEM foils were prepared by an ion-beam thinning technique, using a Gattan-691 precision ion polishing system with liquid-nitrogen cooling. TEM observations of precipitates were performed on selected samples, using a JEM2100F transmission electron microscope operating at 200 kV. The samples prepared for micro-hardness were ground with abrasive paper and polished with 1.5 μm diamond compound. A Vickers hardness tester (FM-800, Hengyi electronic technology, Guangdong, China) was employed to obtain micro-hardness of different samples, operated with a load of 100 gf and holding time of 15 s.

## 3. Results and Discussion

### 3.1. Effect of Pressure on the Microstructure of As-Cast Component

#### 3.1.1. Effect of High Pressure on the Microstructure Characteristics

**Figure 2:**
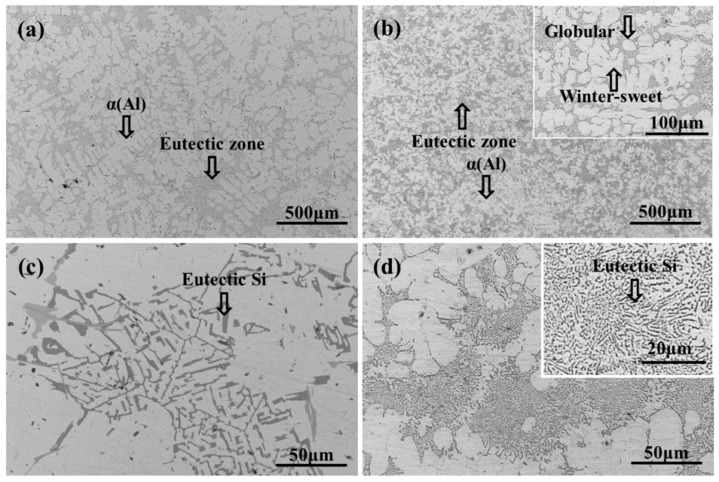
Microstructure of alloy formed by different casting methods: (**a**) and (**c**) alloy formed by gravity casting, (**b**) and (**d**) eutectic Si formed by squeezing casting.

Figure 2 shows the microstructure of the aluminum alloys fabricated by squeezing casting and conventional gravity casting. The microstructure of the gravity casting sample contains the typical coarse dendrite with a secondary dendrite arm spacing of 50–60 μm, as shown in Figure 2a. The fine grains of the squeezing cast alloy shown in Figure 2b indicate that the application of solidification pressure can promote the microstructure refinement. Based on the Clausius–Clapeyron formula as expressed by Equation (1) [19], Ashiri et al. [5] proposed a new version of the equation that can calculate the variation (Δ*T_f_*) of the freezing point (*T_f_*) caused by solidification pressure (*P*), as shown in Equations (2) and (3):

(1) $$\frac{dT}{dP}=\frac{T_{f}\left(V_{L}-V_{s}\right)}{\Delta H_{m}}$$

(2) $$\frac{\Delta T_{f}}{\Delta P}=\frac{T_{f}\left(V_{L}-V_{s}\right)}{\Delta H_{m}}$$

(3) $$\Delta T_{f}=T_{fP}\;-\;T_{f}=\left(\frac{PMT_{fp}}{\Delta H_{m}}\right)\left(\frac{\rho_{L}-\rho_{s}}{\rho_{s}\times \rho_{L}}\right)$$

where *T_fP_* is the freezing point of the alloy under high pressure (*K*), *V_L_* is the specific volume of liquid (cm^3^/g), *Vs* is the specific volume of the solid (cm^3^/g), Δ*H_m_* is the latent heat of solidification (J/mol), *M* is molar mass of the alloy (mol/g), and *ρ_s_* and *ρ_L_* are the densities of the solid and molten alloy under the squeezing pressure (g/cm^3^). They demonstrated that the increment of the solidification temperature is about 6 °C for every 100 MPa pressure increase during solidification of the Al–Si–Mg alloys. Therefore, the solidification temperature can be increased by 8.1 °C corresponding to the pressure of 135 MPa applied in this work. According to the research performed by Li et al. [20], the temperature of the liquid alloy has a decline of around 65 °C during the pouring and mold filling process of the squeezing casting. Based on this conclusion, when the filling process is finished, the melt temperature drops to 615 °C, which is lower than the theoretical freezing point (without solidification pressure) of the alloy used in this study. The under-cooling of the melt is further enhanced by the increment of the freezing point due to the application of the high solidification pressure after the filling process. Thus, the number of nuclei will increase, resulting in the refinement of microstructure.

It is well known that a solid shell will be formed due to fast cooling when the molten alloy touches the internal surface of the mold during the filling process. During the conventional casting process, an air gap between solid shell and mold will appear due to the thermal contraction of the shell which can reduce the heat transfer rate. However, the gap can be eliminated by applied solidification pressure in the squeezing casting process, resulting in the increase of the cooling rate and decrease of time for the grain growth. The refinement of the microstructure is caused by the combination of high under-cooling and improvement of heat transfer rate.

**Figure 3:**
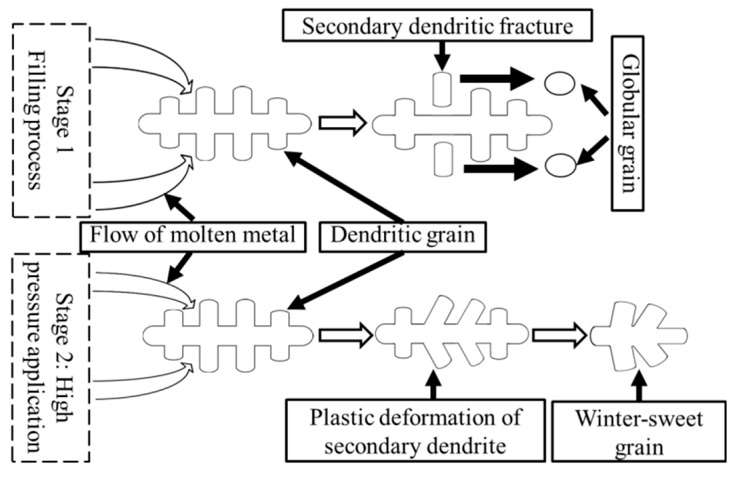
Illustration of fragmentation and plastic deformation of primary α (Al) phase during squeezing casting.

As shown in the upper corner of Figure 2b, the microstructure is composed of the primary α (Al) phases and eutectic composition (α + Si). The primary α (Al) grains of the Al alloy concerned in this work are in spherical, elliptical and petaloid shapes, different from the results obtained by Wang et al. [5] and Ashiri et al. [12], which showed fine dendritic primary α (Al) grains. During the filling process, some dendritic grains formed on the internal surface of the pouring runner and mold. Those dendritic grains would be broken by the intense melt flow resulted from the high filling speed, and the fragments of grains will form the α (Al) phases with the shapes of sphere and short rod. After the filling process, the molten metal containing a certain proportion of solid phase in the mold was immediately applied with a high pressure of 135 MPa. During the subsequent solidification process, a significant volume contraction of the alloy can be caused by the combination of the application of high pressure and the contraction of the alloy. With the application of high pressure, the punch moved forward to promote the further flow of the residual molten metal and activate new feeding mechanisms. The plastic deformation of the secondary dendrite arms could be caused by the re-mobility of the residual molten metal, and lead to the formation of the winter-sweet grains. Figure 3 illustrates the fragmentation process and the plastic deformation process of the primary α phase.

The eutectic Si formed through gravity casting is shown in Figure 2c with the shape of long strips, blocks and needles. However, the shape of the eutectic Si cannot be clearly distinguished in squeezing casting under the same magnification. The enlarged image inserted in Figure 2d reveals the granular and short bar eutectic Si particles with a finer size. During the solidification process under high pressure, the growth of eutectic Si was inhibited by the rapid cooling and some of the preformed eutectic Si may be fragmented by the melt flow resulted from the high pressure.

#### 3.1.2. Effect of High Pressure on the Microhardness

**Figure 4:**
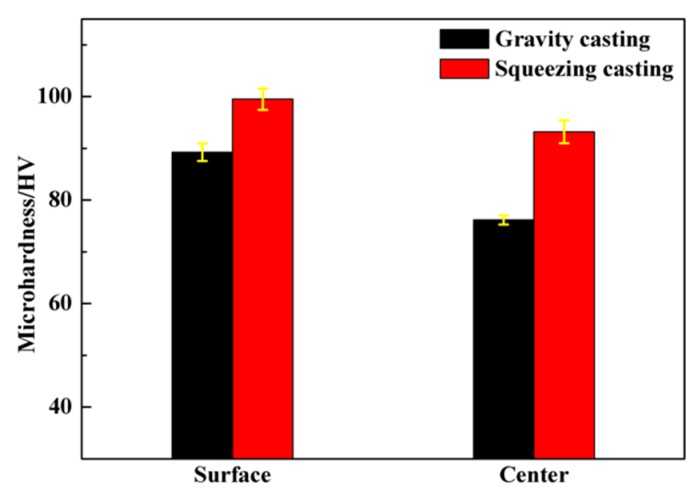
Micro-hardness of the alloy formed by squeezing casting and gravity casting technology.

The micro-hardness of the casting is shown in Figure 4. It can be found that, compared with gravity casting, squeezing casting improved the hardness of the alloy. After squeezing casting, the hardness at the center and the surface of the cast alloy were 93.8 and 100.2 HV, which were increased by 22.4% and 11.3%, respectively. In squeezing casting, the high pressure helped to eliminate most of the defects, such as the blow holes and shrinkage porosity. Meanwhile, the cooling rate was enhanced by the application of high pressure. The diffusion of alloy elements was suppressed due to the rapid cooling which resulted in the increase of Mg, Si and Cu element content in the α (Al) matrix. According to the work of Shercliff et al. [21], the increment (Δ*σ*) of the yield strength caused by solution strengthening can be computed by Equation (4):

(4) $$\Delta \sigma =kC^{2/3}$$

where *k* is a constant in connection with atomic size and elasticity modulus, and *C* is the concentration of the alloy elements. The yield strength can increase significantly with the increase of alloy element content, resulting in the increase of micro-hardness.

Generally, there is a difference in micro-hardness between the center and surface of the cast alloy. The surface layer of the cast alloy is transformed from a solid shell with a faster cooling rate than the center. Therefore, the surface layer can achieve a smaller grain size and higher element content in the α (Al) matrix, resulting in higher microhardness of the surface layer than the cast center. In addition, Figure 4 indicates that the difference of the hardness values between the surface and the center of the casting parts is narrowed from 13.4 HV (gravity casting) to 6.2 HV (squeezing casting). As explained above, the application of high pressure during squeezing casting can help increase the cooling rate and remove the blow holes and the shrinkage porosity, which are mainly distributed inside the cast. This will cause the increase of hardness inside the cast, but has little effect on the hardness of the surface layer, reducing the micro-hardness gap between the central part and the surface layer of the cast. Therefore, squeezing casting makes the material more uniform.

### 3.2. Effect of Solution Treatment on the Microstructure of As-Cast Component

#### 3.2.1. Evolution of Eutectic Si Morphologies during the Solution Treatment Process

**Figure 5:**
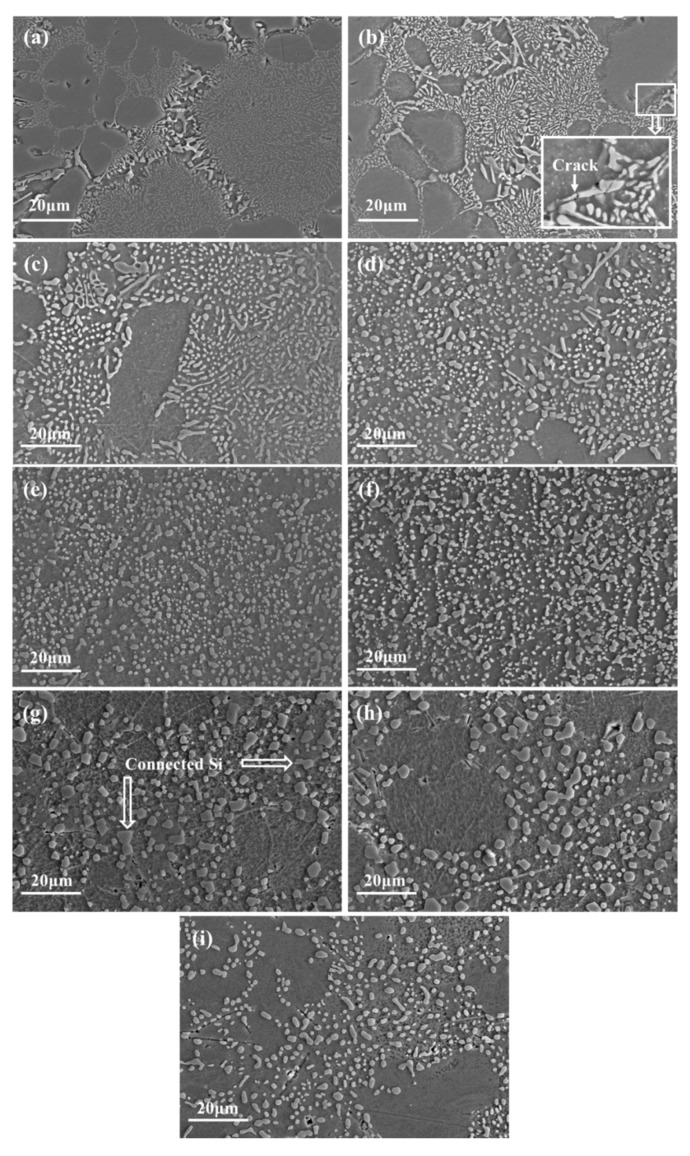
Morphology of eutectic Si under different solution treatment (**a**–**h**: sample formed by squeezing casting; **i**: sample formed by gravity casting): (**a**) 0 min, (**b**) 15 min, (**c**) 30 min, (**d**) 45 min, (**e**) 60 min, (**f**) 120 min, (**g**) 180 min, (**h**) 240 min, (**i**) 120 min.

In order to further study the evolution of the eutectic Si particles, the morphology of eutectic Si is statistically analyzed. Figure 5a–h shows the eutectic Si of the squeezing casting samples under different holding times, ranging from 0 to 240 min, under the temperature of 535 °C. From Figure 5a,b, it can be seen that there are no significant variations in morphology of Si after holding 15 min. However, micro-cracks can be observed at the joint of interconnected Si phase, as shown in the rectangle of Figure 5b. According to the research of Liu et al. [22], the thermal expansion coefficient of the α (Al) phase is about four times that of the Si phase. During the heating or cooling processes, severe stress concentration can be generated between the two phases and leads to the cracking of the brittle Si phase.

With the increase of holding time, spheroidization of the fine Si can be observed obviously, and the long strip or needle-shaped coarse Si phases transform into the small fragments, as shown in Figure 5c,d. During the solidification process, a large number of crystalline defects were formed around the Si particles due to the incompatibility between the eutectic Si and α (Al) phases, which enhanced the diffusion rate of the atom significantly. In addition, the distortion energy of the sharp edge and corner of the Si phases is high due to a higher curvature, which induces more rapid diffusion of atoms than other sites with a lower curvature [23]. More rapid diffusion at the sharp edge causes the spheroidization of Si phases. Fragmentation of the long eutectic Si phases can be also attributed to the rapid diffusion at the concave parts of the strip Si. After solution treatment at 535 °C for 60 min or 120 min, the fine bar and coarse block Si changed into fine spheroidal particles (Figure 5e,f).

When the solution treatment time is further increased to 180 min or 240 min, Si particles started to coarsen. As shown in Figure 5g,h, the average Si particle size is much larger and the coarse Si phases exhibit a facet characteristic with some Si particles connected together (labeled by arrows). In order to reduce the surface energy, dissolving of small Si and growing of large Si are promoted by the diffusion of Si atoms. Liu et al. [22] proved that the facets of coarse Si particles are mostly the {111} Si planes. According to the reference [24], the <112> directions of {111} Si planes are the preferred growth orientation of Si particles. Furthermore, the {111} close-packed surfaces show the lowest surface energy and high atomic binding [25]. Consequently, the growth of coarse Si phases occurred anisotropically with the {111} planes as the external surfaces. In addition, comparing Figure 5f of the squeezing casting specimen with Figure 5i of the gravity casting specimen, it can be found that the sample formed through squeezing casting has a finer Si phases than the specimen made by gravity casting under the same solution treatment of 120 min at 535 °C.

#### 3.2.2. Effect of Solution Treatment on Fe-Rich Intermetallic Phases and the Distribution of Alloy Elements

**Figure 6:**
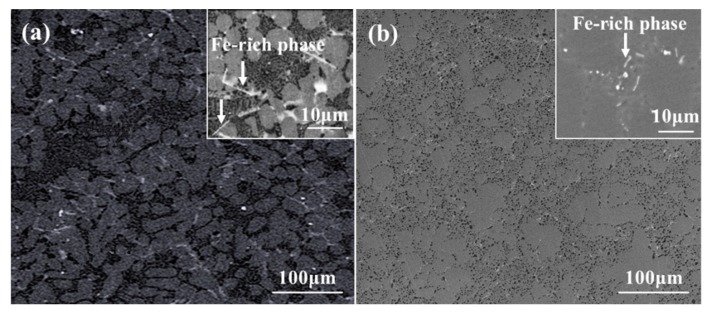
Morphology of the Fe-rich phase of the alloy formed by squeezing casting: (**a**) as-cast sample, (**b**) sample after experienced solution treatment.

Iron is referred to as the most common impurity in aluminum alloys and cannot be eliminated economically. The stress concentration can be induced by the formation of the needle-like Fe-rich intermetallic phase, which reduces the mechanical property. The solid solution treatment can significantly reduce the adverse effect of the needle-like Fe-rich intermetallic phases through improving the morphology of the Fe-rich phases. The morphologies of Fe-rich phases on the polished (without etching) surface of the as-cast and solution-treated samples are shown in Figure 6. Through comparing the Figure 6a,b, it can be suggested that the sharp tips were blunted by the solution treatment. Therefore, the stress concentration caused by Fe-rich phases was relieved, resulting in the mitigation of detrimental effects induced by the Fe-rich phases on mechanical performance.

**Figure 7:**
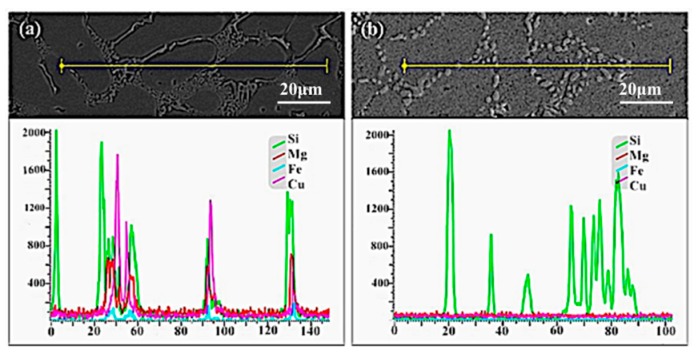
EDS (Energy Dispersive Spectrometer) results of alloy formed by squeezing casting technology (**a**) EDS result of the as-cast alloy; (**b**) EDS result of the alloy after solid solution treatment.

According to Figure 7a, it could be found that numerous alloy elements (Cu and Mg) were concentrated at the grain boundary of the as-cast sample. In order to obtain uniform supersaturated solid solution for ageing treatment, it is necessary to improve the distribution of alloy elements through the solid solution treatment. The EDS (Energy Dispersive Spectrometer) result of squeezing casting sample with solution treatment is shown in Figure 7b, which indicates that the uniform distribution of alloy elements (Cu, Mg) can be obtained through the solution treatment of 120 min at 535 °C. The holding time is shortened by the intermetallic particles’ refinement and improved distribution of alloy elements in squeezing cast samples, compared to the gravity cast which normally requires about 240 min [26].

### 3.3. Effects of the Ageing Process on Hardness and Precipitates

**Figure 8:**
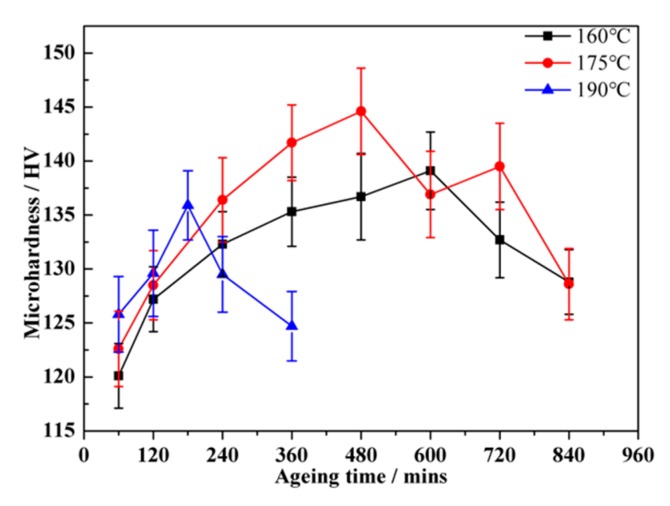
Micro-hardness curves of the alloy after experienced various ageing treatment.

As a typical ageing hardenable alloy, the mechanical properties of the alloy mainly depend on the type, size and density of the precipitates that form in the ageing treatment. The supersaturated solid solution (SSSS) formed through solution treatment provides good conditions for precipitate formation during the subsequent ageing process. The ageing treatment following the solution treatment can result in the formation of precipitates and the increase of the mechanical performance of the alloy. In the present work, the ageing treatment was performed at target temperatures of 160 °C, 175 °C and 190 °C for different periods of time ranging from 60 min to 840 min. The micro-hardness of the α (Al) matrix in the Al alloy was measured corresponding to different ageing treatments. Figure 8 shows that the micro-hardness of specimens increases rapidly at the early stage of the ageing treatment. With the extension of the ageing time, the increase rate declines and the peak ageing time corresponding to the maximum micro-hardness value was obtained for each ageing temperature. When the holding time is longer than the peak ageing time, the micro-hardness of the alloy decreases due to the over-ageing effect. It can be found that the peak ageing time decreases with the increase of the ageing temperature and the highest peak ageing micro-hardness value (144.6 HV) was obtained in the sample subjected to ageing treatment at 175 °C for 480 min.

**Figure 9:**
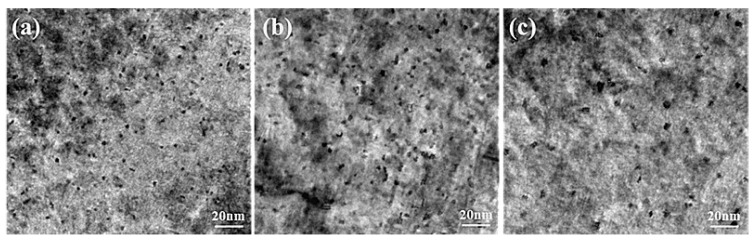
Transmission electron microscopy (TEM) images of the sample after experiencing different ageing treatments: (**a**) 160 °C at 600 min, (**b**) 175 °C at 480 min, (**c**) 190 °C at 180 min.

**Figure 10:**
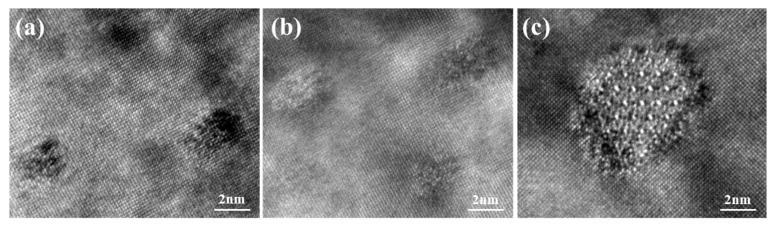
The high-resolution transmission electron microscopy (HRTEM) images of the sample after experienced different ageing treatments: (**a**) 160 °C at 600 min, (**b**) 175 °C at 480 min, (**c**) 190 °C at 180 min.

Figure 9 displays the TEM images of sample peaks aged at 160 °C for 600 min, 175 °C for 480 min and 190 °C for 180 min. The high-resolution transmission electron microscopy (HRTEM) images corresponding to Figure 9, taken from [001] Al zone axes, are presented in Figure 10. It can be seen that dense and fine precipitates distribute uniformly in the α (Al) matrix after ageing at 160 °C for 600 min, as shown in Figure 9a. The majority of precipitates in this sample were coherent with the α (Al) matrix (Figure 10a). The sample aged at 175 °C for 480 min (Figure 9b) possesses slightly larger precipitates and similar number density in the α (Al) matrix, compared with that observed in Figure 9a. However, Figure 10b indicates that most of the precipitates become semi-coherent with the matrix. The coarse precipitates with low number density were distributed in the α (Al) matrix when the sample experienced ageing at 190 °C for 180 min (Figure 9c). Meanwhile, those precipitates become incoherent with the matrix (Figure 10c). In this work, the β phases (Mg2Si) are replaced entirely by the Q phases (Al5Mg8Cu2Si6) since the ratio of Cu/Mg (2.57) is larger than 2.1 [27]. Therefore, the precipitates induced by ageing treatment are composed of GP zones (Guinier Preston zone), the metastable phases of Q phases (Al5Mg8Cu2Si6) and θ phases (Al2Cu). The precipitation sequence can be summarized as follows: SSSS → solute clusters → solute clusters + GP zones → metastable precipitates (Q’ phase and θ’ phase) → equilibrium precipitates (Q phase and θ phase). The GP zones can be regarded as the metastable precipitates in a coherent relation to the α (Al) matrix. During the ageing treatment, Cu atoms dissolved in the α (Al) matrix combine with Mg, Si, and Al atoms to transfer into the Q’ phases in a semi-coherent relation to the α (Al) matrix. The remaining Cu atoms combine with Al to form θ’ phases in a semi-coherent relation to the α (Al) matrix. With the increase of ageing time, metastable precipitates (Q’ phase and θ’ phase) can transfer to equilibrium precipitates (Q phase and θ phase) in an incoherent relation to the α (Al) matrix.

Generally, the primary strengthening mechanism of the AlSiCuMg alloys is precipitate strengthening. The increment of the mechanical properties is associated with the type, size, and density of the precipitates distributed in the α (Al) matrix. After ageing at 160 °C for 600 min, dense and fine precipitates play the critical role for the peak hardness value. In the sample after ageing at 175 °C for 480 min, the particles in a semi-coherent relation to the α (Al) matrix has a stronger inhibition on the dislocation motion than the particles with a coherent relation, resulting in higher hardness. With the increase of the ageing time, the sizes of GP zones and Q’ phases increase and lead to the microhardness decline. According to previous research [28], the formation of the θ’ phase appears later than Q’. Therefore, the number of the θ’ phases could be increased by the ageing time, which eliminates the detrimental effect of the precipitate coarsening through the improvement of the strengthening effect induced by the θ’ phases. The increase of the microhardness (between 600 and 720 min) is caused by the θ’ phases formation. In the sample aged at 190 °C for 180 min, the density of the coarse precipitates is apparently lower. Therefore, the adverse influence on the micro-hardness was introduced and led to the decline of the micro-hardness.

## 4. Conclusions

1. The application of high pressure during squeezing casting could increase the under-cooling and cooling rate, resulting in refined grains of the α (Al) matrix and the eutectic Si phases, as well as the formation of the winter-sweet shaped grains due to the plastic deformation of the dendrites.

2. In the squeezing casting process, the defects were eliminated. Meanwhile the alloy element content was also increased by rapid cooling induced by application of high pressure. The micro-hardness of the squeezing cast sample was over 22% higher than that of the gravity cast.

3. The effect of high pressure on the defects and the cooling rate in the center part was more obvious than the surface layer, which led to the reduction of the micro-hardness difference between the surface and center of the squeezing cast.

4. The maximum isothermal holding time of solution treatment at 535 °C was 120 min. If longer than 120 min, the coarsening and coalescence of the eutectic Si particles will occur.

5. During the ageing treatment, a large number of fine precipitates formed, which increased the microhardness through inhibiting the slip motion of dislocation. A maximum (peak) micro-hardness value was obtained for each ageing temperature, and the highest peak value was achieved at 175 °C for 480 mins.

## Figures and Tables

**Table 1:** Chemical composition of the AlSi9CuMg aluminum alloy (wt.%).

Si	Cu	Mg	Fe	Al
9.0	0.85	0.33	0.20	Bal.
